# Comparison of multivariable methods for determining cutpoints of biomarkers in the context of survival time analyses: A simulation study with practical applications to survival data

**DOI:** 10.1371/journal.pone.0338425

**Published:** 2025-12-05

**Authors:** Jan Porthun, Andreas Wienke

**Affiliations:** 1 Norwegian University of Science and Technology, Gjøvik, Norway; 2 Institute of Medical Epidemiology, Biostatistics, and Informatics, Martin-Luther-University Halle-Wittenberg, Halle, Germany; Indiana University Indianapolis, UNITED STATES OF AMERICA

## Abstract

**Introduction:**

Survival time models are commonly employed in medicine and health sciences when analysing data. In these time-to-event analyses, it is often necessary to dichotomise variables that are metrically measured. One example could be to assign patients to different risk groups based on an occurring event. Besides univariable methods, multivariable approaches also exist for establishing cutpoints. Up to now, these multivariable approaches have hardly been investigated.

**Methods:**

Using a Monte Carlo simulation study, we analysed eight multivariable methods from the literature to establish a cutpoint of a biomarker in the context of a semiparametric Cox regression model. The methods are the following: maximising the chi-square statistic, maximising the chi-square statistic with a split-sample approach, maximising the c-index using either the AddFor- or Genetic algorithm, maximising the concordance probability estimator (CPE) with the AddFor- or Genetic algorithm, and minimising the Akaike information criterion (AIC). We compared these methods with each other and in addition with the univariable log-rank minimum p-value approach. The simulation parameters analysed included the cutpoint’s distance from the biomarker’s median, sample size, total censoring, censoring before the end of the follow-up time (drop-outs), and the survival time distribution. Bias and empirical standard error were used as the primary performance measures. Furthermore, each method is illustrated using two practical data examples.

**Results:**

All analysed methods are biased towards the biomarker’s median. Multivariable methods that estimate the cutpoint by using the lowest AIC or the maximum of the chi-square statistic have the lowest bias and empirical standard error in most simulation scenarios. The difference in bias between the methods based on maximising the c-index or maximising the CPE is minimal. Regardless of the distribution used (Weibull, Gompertz, or exponential), the respective bias shows similar dependencies on the simulation parameters.

**Conclusions:**

Multivariable methods to estimate a biomarker’s cutpoint in survival time analyses using the Cox regression model may represent a good alternative to univariable methods. Our simulation has shown that methods maximising the chi-square statistic or minimising the AIC, respectively, perform better than the univariable method using the minimum p-value approach and outperform multivariable methods based on the c-index or CPE.

## Introduction

Survival models are regularly used in medical research as well as in research within the field of health sciences [1]. Their usage refers not only to analyses concerning the survival of patients over a certain period, but also to other time-to-event analyses. One example could be the time span of release from the hospital after surgery; another the time period between an intervention and the absence of symptoms afterwards. The semiparametric Cox proportional hazards model is often applied here [1,2]. It is not bound to specific distribution parameters, and estimated hazard ratios (HRs) allow reliable conclusions to be drawn, provided that the effect remains constant over time [3]. In relation to survival models, cutpoints of metrical biomarkers are regularly established [4–6]. Cutpoints are used, for instance, to divide patients into groups with different survival expectations depending on the levels of a specific biomarker [7]. This is utilised in clinical studies in which stratification is based on covariates [8,9]. In the literature, the terms ‘threshold’ or ‘changepoint’ are also used instead of cutpoint [10–12]. Cutpoints are also important in everyday clinical work. The interpretation of metric biomarkers is often done via established cutpoints. One of the most known illustrations is the category for which systolic and diastolic blood pressure is interpreted. According to the Guideline for the Prevention, Detection, Evaluation, and Management of High Blood Pressure in Adults of the American College of Cardiology and the American Heart Association, a systolic blood pressure <120 mmHg combined with a diastolic blood pressure <80 mmHg is considered ‘normal’ [13]. An exemplification of the formation of cutpoints in the context of survival time analyses is the study of Otten et al. with advanced-stage non-small cell lung cancer patients. They investigated the prognostic potential of immune checkpoint inhibitor clearance and determined the cutpoint for nivolumab clearance at ≥7.3 mL/h at first dose. Patients whose nivolumab clearance was below the cutpoint had a higher risk of death [14].

The dichotomisation or stratification of metric variables with the help of cutpoints is associated with a loss of information and power in the context of statistical analyses. Therefore, it should always be carefully weighed up, whether the formation of cutpoints is necessary [15,16].

As part of our research, we were preoccupied with the question of estimating cutpoints in the context of survival analyses using the Cox regression model. We focused on application areas, where data sets of up to 1000 patients are available. In these cases, AI-based analyses have been only helpful to a limited extent due to the small number of subjects. In the following, we always refer to the scenario, that a cutpoint of a biomarker is determined in the context of survival analysis using Cox regression for right-censored data.

Different methods are suggested for this purpose in the literature. One classic method uses the biomarker’s median or quartile boundaries as a cutpoint [17]. Another frequently applied method is the minimum p-value approach [18]. Two subgroups are formed at all potential cutpoints of the biomarker. Based on these two groups, a log-rank test is executed. The value of the biomarker, at which the p-value of the log-rank test is the smallest, is used as the cutpoint. This approach considers the observation time and status in addition to the distribution of the biomarker.

Studies published from 2003 to 2022 dealt with the formation of cutpoints in a multivariable setting within the framework of Cox regression. The researchers who published these studies suggested to consider not only the biomarker to be dichotomised but also other relevant covariates [19–22]. Thus, the determination of the cutpoint is carried out within the framework of a multivariable setting. Different procedures have been proposed for this. The authors of these multivariable methods argue that multivariable methods are superior to univariable methods that only consider the biomarker itself. They explain this by stating that a more precise estimate of the true cutpoint is expected. This is the case because the model includes other variables that contribute to the variability of the dependent variable [22]. All multivariable methods have in common that the biomarker, for which a cutpoint is to be determined, is included with other cofactors in a multivariable Cox regression model. Based on these Cox regression models, different parameters are used to determine and choose the cutpoint within the framework of the methods proposed in the literature. The different methods are listed below, having been added with abbreviations.

### Method A) Maximising the chi-square statistic with a twofold cross-validation approach (max *χ*^*2*^)

Separate Cox regression models are calculated for all possibilities to dichotomise the biomarker under consideration. The cutpoint is used, for which the Cox regression model’s chi-square statistic assumes the largest value. This method is a modification of the approach described by Mazumdar et al. The corresponding p-values and hazard ratios are determined through a twofold cross-validation [22].

### Method B) Maximising the chi-square statistic with a split-sample approach (max *χ*^*2*^ split-sample)

This method also determines the cutpoint based on the maximum chi-square value. However, only half of the data set is used for this purpose. The p-values and hazard ratios are estimated with the other half of the dataset [22].

### Method C) Maximising the c-index with the AddFor- or Genetic algorithm (c-index AddFor/ Genetic)

The cutpoint, for which the c-index takes the largest value, is chosen. Either the AddFor (method C1) or genetic algorithm (method C2) can be used [19].

### Method D) Maximizing of the concordance probability estimator (CPE) with the AddFor- or Genetic algorithm (CPE AddFor/ Genetic)

The maximum of the CPE is the basis for determining the cutpoint. When using the CPE, either the AddFor (method D1) or genetic algorithm (method D2) can be used [19].

### Method E) Minimum of the AIC (min AIC)

The cutpoint of the biomarker is the value, at which the AIC has its lowest value for the respective Cox regression model. The dichotomised variable can be included in the Cox regression model either as a covariate (method E1) or as a strata variable (method E2) [21].

The authors of the described methods (A, B, C1, C2, D1, D2, E1, and E2) have investigated them in the context of simulation studies [19,21,22]. Mazumdar et al. compared methods A and B with each other; additionally with the univariable minimum p-value approach. They conclude that the multivariable method A (max *χ*^*2*^) is more efficient in finding the cutpoint than the univariable method [22]. In the context of the multivariable methods, they prefer the cross-validation approach to estimate HRs and p-values and not method B (max *χ*^*2*^ split-sample). In the latter only half of the dataset is used to determine the cutpoint. The authors generated the survival times utilising an exponential distribution. Barrio and colleagues compared the Genetic algorithm-based methods C2 and D2 [19]. They did not examine the AddFor algorithm. Based on their simulation study, these authors recommend using either the c-index or CPE for less than 50% censoring rates and the c-index for higher censoring rates. In their simulation, the survival times were generated using a Weibull distribution.

According to our research, no study has been published concerning the determination of a cutpoint for a metric biomarker within the context of survival analyses, wherein various variants of multivariable methods (A to E2) are compared to one another. Furthermore, in the existing simulation studies, the generated survival times are solely based on one specific distribution: either an exponential or a Weibull distribution.

Our main objective is to compare all methods identified in the literature for determining a cutpoint based on a metric biomarker within a Monte Carlo simulation study. These include the methods previously described (A, B, C1, C2, D1, D2, E1 and E2). We aim to ascertain whether it is feasible to identify a cutpoint using these methods. Additionally, we will compare these methods to the univariable approach using the minimum p-value method (method F). We also illustrate all methods on two real clinical data examples.

## Materials and methods

The simulation study follows the recommendations by Morris et. al. [23].

### Simulation design

**Fig 1:**
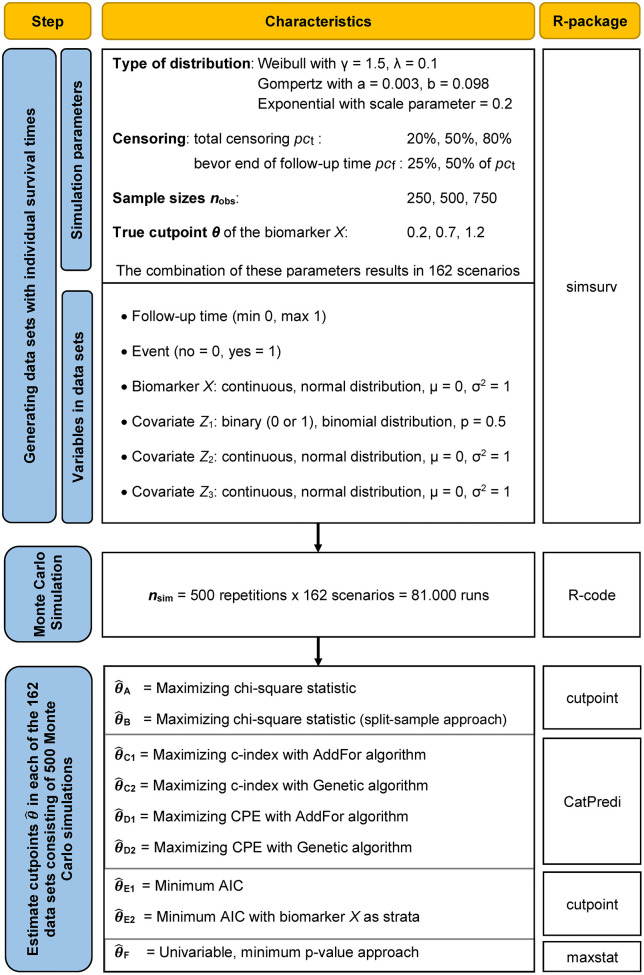
Flow diagram of the simulation study and software used. AIC; Akaike information criterion; cp, cutpoint; CPE, concordance probability estimator; n_obs_, sample size; pc_t_, total censoring; pc_f_, censoring before end of follow-up time in percent of total censoring (pc_t_); n_sim_, repetitions; θ, true cutpoint.

**Fig 2:**
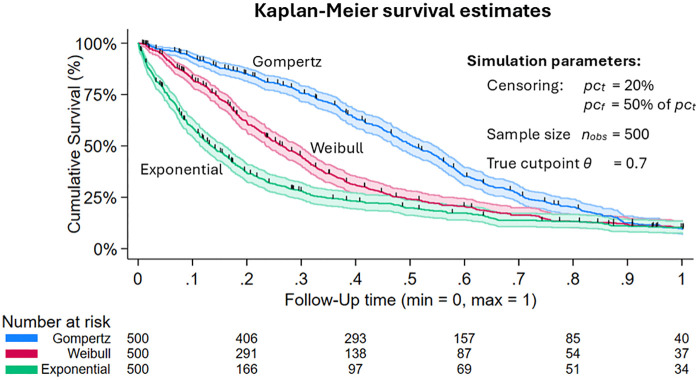
Examples of Kaplan-Meier curves with 95% CI for the three types of distributions. Gompertz distribution with a = 0.003, b = 0.098; Weibull distribution with γ = 1.5, λ = 0.1; Exponential distribution with scale parameter = 0.2; CI, Confidence interval; n_obs_, sample size, pc_t_, total censoring; pc_f_, censoring before end of follow-up time in percent of total censoring (pc_t_); θ, true cutpoint.

The entire simulation was performed for a Cox proportional hazard model with right-censored data. The corresponding model is


h(t, X, Z`; βX , β` )=h0(t) exp {βX X + β` Z` }, (1)

with h_0_*(t)* as baseline hazard function at time *t* and the predictor variables *X* and *Z`*. For the simulation, right-censored survival times *T*_*S*_ with max = 1 were generated with the help of the R-Package Simsurv [24]. According to Bender et al., Weibull and Gompertz distributions were also considered in addition to exponential distribution [25]. For the associated formulas to determine *T*_*S*_, the detailed descriptions are referred to [25]. Four variables were included in the model (Formula 1). The continuous biomarker *X*, a binary covariate *Z*_*1*_, and two continuous covariates *Z*_*2*_ and *Z*_*3*_ (See Fig 1). The associated betas are: *β*_*X*_ = ln(3), *β*_*Z1*_ = ln(2), *β*_*Z2*_ = ln(0.5) and *β*_*Z3*_ = ln(2). A true cutpoint *θ* was used to dichotomise the biomarker *X* in a binary variable *X*_*D*_ with *X*_*D*_* *= 0 if *X *≤ *θ* and *X*_*D*_ = 1 if *X* > *θ*. The individual censoring times *C* have a uniform distribution *U*_*[0,1]*_. The parameter *pc*_*t*_ was used to control the total number of censoring proportion. To distinguish between administrative censoring before the end of the follow-up time and at the end of the follow-up time, the parameter *pc*_*f*_ is used. The final follow-up times are *T* = min(*T*_*S*_, *C*). An overview of the simulation parameters can be found in the flow diagram (Fig 1). The combination of all these parameters results in a total of 162 simulation scenarios. Fig 2 shows three examples of generated censored survival times (Fig 2).

The generated datasets contain the variables follow-up time, event (no = 0, yes = 1), the biomarker *X* in its original continuous form, and the covariates *Z*_*1*_, *Z*_*2,*_ and *Z*_*3*_. All datasets are employed to estimate the true cutpoint *θ* of the biomarker X using the multivariable methods mentioned above (A, B, C1, C2, D1, D2, E1, and E2) as well as the univariable minimum p-value approach (method F) (see Table 1). The estimated cutpoints are referred to as ${\hat{\theta }}_{𝐀}$, ${\hat{\theta }}_{𝐁}$, ${\hat{\theta }}_{𝐂1}$, ${\hat{\theta }}_{𝐂2}$, ${\hat{\theta }}_{𝐃1}$, ${\hat{\theta }}_{𝐃2}$, ${\hat{\theta }}_{𝐄1}$, ${\hat{\theta }}_{𝐄2}$ and ${\hat{\theta }}_{𝐅}$ – following the methods’ names and introduced above (see Table 1 and Fig 1).

**Table 1:** Overview and brief description of the simulation methods used.

Simulation method	Estimated cutpoints	Short description
A	${\hat{\theta }}_{𝐀}$	Maximising the chi-square statistic involves selecting the cutpoint that yields the highest chi-square from a multivariable Cox model. The statistic is computed for all potential cutpoints of the variable, and the optimal one maximises the chi-square. [22]
B	${\hat{\theta }}_{𝐁}$	Maximising chi-square statistic on half the data set (split-sample approach). [22]
C1	${\hat{\theta }}_{𝐂1}$	The c-index, also known as Harrell’s C, serves as a global measure of concordance probability. The optimal cutpoint is the value where the c-index reaches its maximum. For this, the AddFor algorithm is used. [19]
C2	${\hat{\theta }}_{𝐂2}$	Maximising Harrell’s C (c-index) using the Genetic algorithm. [19]
D1	${\hat{\theta }}_{𝐃1}$	Maximising the concordance probability estimator (CPE) using the AddFor algorithm. This method determines the cutpoint at which the CPE attains its maximum value. [19]
D2	${\hat{\theta }}_{𝐃2}$	Maximising the concordance probability estimator (CPE) using the Genetic algorithm. [19]
E1	${\hat{\theta }}_{𝐄1}$	Minimum AIC. The cutpoint is the value at which the Akaike information criterion (AIC) reaches its minimum in the multivariable Cox model. To determine this, the metric variable is dichotomised at all possible cutpoints. The resulting binary variable replaces the original continuous variable as a covariable in the model. [21]
E2	${\hat{\theta }}_{𝐄2}$	Minimum AIC, with the biomarker as strata. The procedure is similar to that of method E1. However, the dichotomised variable is utilised as a strata variable in the Cox regression model. [21]
F	${\hat{\theta }}_{𝐅}$	Minimum p-value approach (univariable). A systematic cutpoint search is used in a univariable setting to determine the cutpoint, which is related to the smallest p-value of the log-rank test. [18]

The table presents the acronyms of various simulation methods used, along with a brief description and their reference numbers.

The respective cutpoints $\hat{\theta }$ for the methods C1, C2, D1, and D2 are estimated using the R-package CatPredi [26]. For the calculation of the cutpoint ${\hat{\theta }}_{𝐅}$ of the univariate method (minimum p-values approach), the R package maxstat, was used [27]. Cutpoints for methods A, B, E1, and E2 were estimated using the R package cutpoint [28]. The Cox regression models were calculated for all possible variants to dichotomise the biomarker. The covariates Z_1_, Z_2_, and *Z*_*3*_ are part of the Cox regression models. ${\hat{\theta }}_{𝐀}$ and ${\hat{\theta }}_{𝐁}$ are estimated from the Cox regression model with the highest chi-square statistic. For method B, only half of the data set was included. In the context of methods E1 and E2, the corresponding cutpoints ${\hat{\theta }}_{𝐄1}$ and ${\hat{\theta }}_{𝐄2}$ were estimated from this Cox regression model with the lowest AIC. For method E1, *X*_*D*_ was a variable in the Cox regression model. For method E2, *X*_*D*_ was used as a strata variable. The performance metrics – derived from the simulations – are presented in both tabular and graphical formats. In addition to boxplots, we utilised a nested loop plot for our visual representation [29].

### Performance measures

We assessed the bias, empirical standard error (EmpSE), mean squared error (MSE), and relative precision gain versus the method with the lowest EmpSE. The corresponding Monte Carlo Standard errors (MCSEs) were determined for all performance measures. The estimated performance measures are defined as follows:


$$\begin{array}{c} \text{Bias }=\frac{\text{1}}{n_{\text{sim}}}\;\sum_{i=\text{1}}^{n_{\text{sim}}}{\hat{\theta }}_{i}-\;\theta . \end{array}$$

$$\begin{array}{c} \text{EmpSE }=\sqrt{\frac{\text{1}}{n_{\text{sim}}-\text{1}}\;\sum_{i=\text{1}}^{n_{sim}}{\left({\hat{\theta }}_{i}-\overset{―}{\theta }\right)}^{\text{2}}}. \end{array}$$

$$\text{Relative }\%\text{ increase in precision }(\text{e}.\text{g}.,\text{ method A vs method B})=\text{1}\text{0}\text{0}\left({\left(\frac{\hat{{EmpSE}_{A}}}{\hat{{EmpSE}_{B}}}\right)}^{\text{2}}-\text{1}\right).$$

$$\begin{array}{c} \text{MSE }=\frac{\text{1}}{n_{\text{sim}}}\;\sum_{i=\text{1}}^{n_{sim}}{\left({\hat{\theta }}_{i}-\;\theta \right)}^{\text{2}}. \end{array}$$

For details on the estimates of the performance measures and their Monte Carlo Standard Error, see [23]. Our most important performance measures are the bias and the EmpSE. Barrio and colleagues, who investigated the methods C2 and D2 in their simulation study, reported standard deviations (SDs) ranging from 0.010 to 0.096 for the means of their cutpoint estimates [19]. Following the practical example of Morris et al. [23], we also have decided that the Monte Carlo SE of the bias should be lower than 0.005. Applying the formula in the same way as Morris et al. for calculating *n*_*sim*_ and using the maximum SD of Barrio et al. (SD = 0.096), we get a required number of simulations *n*_*sim*_ of 369. As we do not know the SDs for the other methods, we have opted for a conservative approach and set *n*_*sim*_ = 500.

## Results

In the 81,000 datasets (500 simulations x 162 scenarios), a maximum of 60 values for each method is missing for $\hat{\theta }$. In these cases, no cutpoints were found using the R package CatPredi. The missing ones are distributed among the methods as followed: C1: 19, C2: 48, D1: 18, D2: 60, meaning 0.022 to 0.074 percent per method. Methods A and E1 yield similar results concerning the performance measures, except for minimal differences (Tables 2 and 3). Therefore, the results for method E1 are presented in the following, but not additionally the results for method A.

**Table 2:** Performance measures of all simulation scenarios with their Monte Carlo standard error in parentheses.

Performance Measure	True cutpoint *θ*	Method to estimate the cutpoint $\hat{\theta }$								
		$\hat{\theta }$_A_ max *χ*^*2*^	$\hat{\theta }$_B_ max *χ*^*2*^ split sample	$\hat{\theta }$_C1_ c-index AddFor	$\hat{\theta }$_C2_ c-index Genetic	$\hat{\theta }$_D1_ CPE Addfor	$\hat{\theta }$_D2_ CPE Genetic	$\hat{\theta }$_E1_ min AIC	$\hat{\theta }$_E2_ min AIC with Strata	$\hat{\theta }$_F_ min p-Value
**Bias** **(distance to true cutpoint *θ*)**	**0.2**	**−0.0005** (2.3e-05)	−0.0037 (4.7e-05)	−0.0107 (5.0e-05)	−0.0091 (4.5e-05)	−0.0111 (9.2e-05)	−0.0137^*^ (8.7e-05)	**−0.0005** (2.3e-05)	0.0104 (0.0002)	−0.0093 (5.7e-05)
	**1.2**	**−0.0207** (0.0002)	−0.1058 (.0007)	−0.1480 (0.0009)	−0.1425 (0.0008)	−0.1463 (0.0009)	−0.1494 (0.0009)	**−0.0206** (0.0002)	−1.1026^*^ (0.0004)	−0.0546 (0.0003)
**Empirical standard error** **(EmpSE)**	**0.2**	**0.0038** (1.7e-05)	0.0078 (3.3e-05)	0.0082 (3.5e-05)	0.0073 (3.2e-05)	0.0150 (6.5e-05)	0.0143 (6.1e-05)	**0.0038** (1.7e-05)	0.0260^*^ (0.0001)	0.0094 (4.0e-05)
	**1.2**	**0.0297** (0.0001)	0.1119 (0.0005)	0.1420 (0.0006)	0.1368 (0.0006)	0.1525^*^ (0.0007)	0.1525^*^ (0.0007)	**0.0297** (0.0001)	0.0586 (0.0003)	0.0547 (0.0002)
**Mean squared error (MSE)**	**0.2**	**1.5e-05** (1.5e-07)	7.4E-05 (1.0e-06)	0.0002 (1.9e-06)	0.0001 (1.4e-06)	0.0004 (5.4e-06)	0.0004 (5.8e-06)	**1.5e-05** (1.5e-07)	0.0008^*^ (8.0e-06)	0.0002 (2.3e-06)
	**1.2**	**0.0013** (1.9e-05)	0.0237 (0.0002)	0.0421 (0.0004)	0.0390 (0.0004)	0.0447 (0.0005)	0.0456 (0.0005)	**0.0013** (1.9e-05)	1.2192^*^ (0.0008)	0.0060 (6.3e-05)
**Relative precision gain vs method E1 (min AIC)**	**0.2**	**0** (0)	−75.57 (0.24)	−77.98 (0.27)	−72.65 (0.33)	−93.49 (0.07)	−92.76 (0.08)	Reference	−97.82^*^ (0.02)	−83.19 (0.16)
	**1.2**	**0.08** (0.01)	−92.95 (0.03)	−95.62 (0.02)	−95.28 (0.02)	−96.20^*^ (0.02)	−96.20^*^ (0.02)	Reference	−74.26 (0.28)	−70.49 (0.13)

The table shows the performance measures with their corresponding Monte Carlo Standard Error (MCSE) in parentheses. The smallest value per row is marked in bold, whereas the highest is marked with an asterisk (*).

**Table 3:** Bias for the Weibull distribution.

True cutpoint *θ*	*n*_*obs*_	Total censoring *pc *_*t*_	Method to estimate the cutpoint $\hat{\theta }$								
			$\hat{\theta }$_A_ max *χ*^*2*^	$\hat{\theta }$_B_ max *χ*^*2*^ split-sample	$\hat{\theta }$_C1_ c-index AddFor	$\hat{\theta }$_C2_ c-index Genetic	$\hat{\theta }$_D1_ CPE AddFor	$\hat{\theta }$_D2_ CPE Genetic	$\hat{\theta }$_E1_ min AIC	$\hat{\theta }$_E2_ min AIC with Strata	$\hat{\theta }$_F_ min p-Value
**0.2**	**250**	**0.2**	**−0.0044**	−0.0105	−0.0191	−0.0181	−0.0110	−0.0151	**−0.0044**	−0.0258^*^	−0.0212
		**0.8**	0.0083	**0.0046**	−0.0319	−0.0284	−0.0455	−0.0418	0.0083	0.0577^*^	−0.0084
	**500**	**0.2**	**0.0003**	−0.0038	−0.0091	−0.0081	−0.0083	−0.0100^*^	**0.0003**	0.0093	−0.0079
		**0.8**	−0.0071	−0.0515	−0.0775	−0.0716	−0.0902	−0.0931	**−0.0069**	−1.1090^*^	−0.0307
	**750**	**0.2**	−0.0010	**−0.0006**	−0.0022	−0.0021	0.0008	−0.0042	−0.0010	−0.0091^*^	−0.0051
		**0.8**	0.0014	**−0.0006**	−0.0054	−0.0044	−0.0085	−0.0091	0.0014	0.0285^*^	−0.0047
**0.7**	**250**	**0.2**	−0.0023	−0.0227	−0.0114	−0.0126	−0.0116	−0.0152	**−0.0022**	−0.5346^*^	−0.0142
		**0.8**	**−0.0034**	−0.0743	−0.0814	−0.0803	−0.1257	−0.1273	**−0.0034**	−0.2037^*^	−0.0351
	**500**	**0.2**	**−0.0031**	−0.0040	−0.0126	−0.0143	−0.0051	−0.0097	**−0.0031**	−0.5642^*^	−0.0122
		**0.8**	**0.0025**	−0.0151	−0.0181	−0.0131	−0.0343	−0.0360	**0.0025**	−0.1899^*^	−0.0098
	**750**	**0.2**	−0.0008	−0.0030	−0.0049	−0.0060	**0.0006**	−0.0045	−0.0008	−0.5783^*^	−0.0063
		**0.8**	**−0.0004**	−0.0066	−0.0094	−0.0053	−0.0167	−0.0172	**−0.0004**	−0.1679^*^	−0.0083
**1.2**	**250**	**0.2**	**−0.0350**	−0.1599	−0.2187	−0.2149	−0.1798	−0.1785	**−0.0350**	−1.1555^*^	−0.1018
		**0.8**	**−0.0921**	−0.3233	−0.4321	−0.4327	−0.4840	−0.4957	**−0.0921**	−1.0086^*^	−0.1825
	**500**	**0.2**	**−0.0093**	−0.0169	−0.0379	−0.0367	−0.0233	−0.0272	**−0.0093**	−1.1675^*^	−0.0236
		**0.8**	**−0.0035**	−0.0818	−0.1226	−0.1116	−0.1808	−0.1810	**−0.0035**	−1.0459^*^	−0.0411
	**750**	**0.2**	**−0.0042**	−0.0085	−0.0227	−0.0253	−0.0101	−0.0169	**−0.0042**	−1.1651^*^	−0.0132
		**0.8**	**−0.0025**	−0.0207	−0.0762	−0.0649	−0.0713	−0.0886	**−0.0025**	−1.0531^*^	−0.0242

*n*_*obs*_, number of observations (for example, number of patients); *pc*_*t*_, total censoring in percent. The smallest bias per row is marked in bold, whereas the highest is marked with an asterisk (*).

### Bias

**Fig 3:**
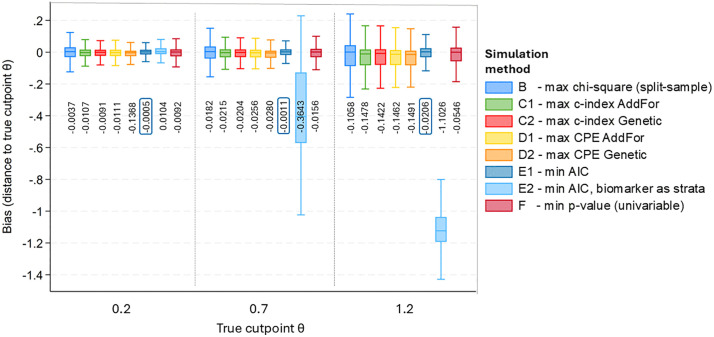
Bias shown by method categorised according to the three true cutpoints θ. Boxplot without outliers. The estimates shown are the means of the bias, categorised after the three true cutpoints θ used in the simulation (0.2, 0.7, 1.2). The smallest values in each group are framed. Method A is not shown because the results are identical to method E1.

**Fig 4:**
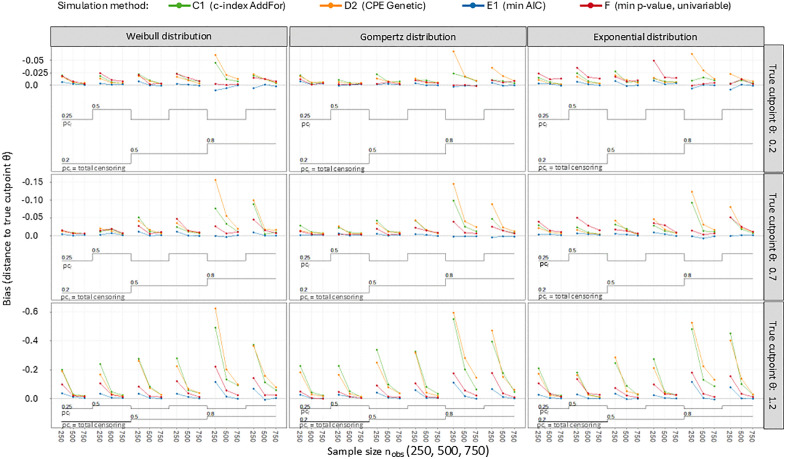
Hybrid nested loop plot considering all 162 simulation scenarios for four simulation methods. A combination of trellis plot and nested loop plot showing six scenarios per layer: pc_t_, = total censoring in percent (0.2, 0.5, 0.8); pc_f_, = censoring before end of follow-up time in percent of total censoring (0.25, 0.50).

**Fig 5:**
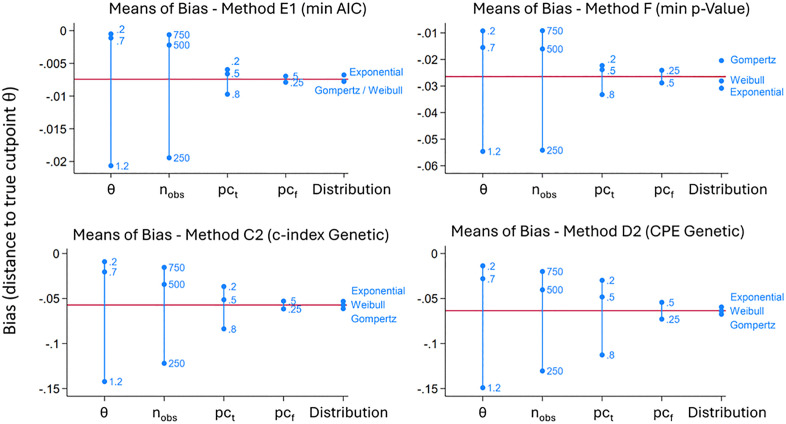
Means of bias for four simulation methods depending on simulation parameters. θ, rue cutpoint (0.2, 0.7, 1.2); n_obs_, sample size (250, 500, 750); pc_t_, total censoring in percent (0.2, 0.5, 0.8); pc_f_, censoring before end of follow-up time in percent of total censoring (0.25, 0.50).

All methods – according to the true cutpoints *θ* – apart from one exception, have a bias with a negative sign (Fig 3). This corresponds to a tendency towards the median of biomarker *X*. Method E1 shows the lowest bias in most scenarios, regardless of the true cutpoint. All methods have a stronger bias, depending on how far the true cutpoint θ is from the median (Table 2 and Fig 5). This is mostly distinctive in method E2. The AddFor algorithm and the Genetic algorithm each have a different bias for the methods C1, C2, and D1, D2. However, neither of these algorithms is associated with lower bias in general. (Tables 2 and 4). Table 3 shows the bias values separated by the values for *θ, n*_*obs*_, and *pc*_*t*_ (Table 3). In three scenarios, method B (max *χ*^*2*^ split sample) has a lower bias than method E1. In one scenario, it is method D1 (CPE AddFor) (Table 3). Regardless of the distribution used (Weibull, Gompertz, or exponential), there are similar dependencies of the respective bias on the simulation parameters (Figs 4 and 5, Table 4). For all methods, the bias increases, the lower the sample size *n*_*obs*_ and the greater the total censoring rate *pc*_*t*_ is (Figs 4 and 5). There is also a larger bias, if *n*_*obs*_ = 250 or *n*_*obs*_ = 500 and simultaneously *pc*_*t*_ and *pc*_*f*_ are 0.8 and 0.25 (Fig 4 and Table 4). For censoring before the end of follow-up time in percent of total censoring (*pc*_*f*_), there are minor effects on the bias depending on the parameters used for *pc*_*f*_ in the simulation (Fig 5). If the methods are sorted according to the lowest to highest bias, the order for *θ* = 0.2 is: E1, B, C2, F, E2, C1, D1, and D2 (Table 2). In the case of *θ *= 1.2, the order is different: E1, F, B, C2, D1, C1, D2, and E2. Table 4 shows the bias for all simulation parameters for method E1 (Table 4). The lowest absolute bias for method E1 is 0.0001, and the largest is found at *n*_*obs*_ = 250, *pc*_*t*_ = 0.8, *pc*_*f*_ = 0.25 with 0.1164. Table 4 also shows that for method E1, there are no particularly low or high bias values in any of the distributions used (Table 4).

**Table 4:** Bias for the method E1 (min AIC).

*n*_*obs*_	*pc*_*t*_	*pc*_*f*_	Weibull distribution		Gompertz distribution		Exponential distribution	
			*θ* = 0.2	*θ* = 1.2	*θ* = 0.2	*θ* = 1.2	*θ* = 0.2	*θ* = 1.2
**250**	**0.2**	**0.25**	−0.0059	−0.0366^*^	−0.0077	−0.0276	**−0.0031**	−0.0259
		**0.50**	−0.0030	−0.0334^*^	**0.0016**	−0.0275	−0.0066	−0.0294
	**0.5**	**0.25**	−0.0074	−0.0354	**−0.0016**	−0.0429^*^	−0.0078	−0.0338
		**0.50**	**−0.0023**	−0.0354	−0.0037	−0.0600^*^	−0.0087	−0.0253
	**0.8**	**0.25**	0.0104	−0.1164^*^	**0.0033**	−0.1099	0.0068	−0.1156
		**0.50**	0.0063	−0.0677	**−0.0045**	−0.0655	0.0095	−0.0785^*^
**500**	**0.2**	**0.25**	−0.0023	−0.0112^*^	**−0.0011**	−0.0029	−0.0021	−0.0038
		**0.50**	−0.0012	−0.0074	**−0.0001**	−0.0116^*^	−0.0018	−0.0034
	**0.5**	**0.25**	**0.0007**	−0.0041	−0.0025	−0.0070^*^	0.0017	0.0031
		**0.50**	−0.0012	−0.0129^*^	**0.0002**	−0.0034	−0.0014	−0.0042
	**0.8**	**0.25**	0.0066	−0.0140	0.0015	−0.0161^*^	**−0.0002**	−0.0056
		**0.50**	**−0.0008**	0.0070	0.0014	−0.0126^*^	−0.0012	−0.0024
**750**	**0.2**	**0.25**	**−0.0005**	−0.0045^*^	−0.0029	−0.0018	0.0013	−0.0033
		**0.50**	−0.0016	−0.0038	−0.0015	−0.0041^*^	**0.0004**	−0.0031
	**0.5**	**0.25**	0.0013	−0.0030^*^	−0.0008	−0.0013	0.0008	**−0.0004**
		**0.50**	0.0012	−0.0021	**0.0005**	−0.0037^*^	−0.0037^*^	−0.0022
	**0.8**	**0.25**	**0.0003**	**−0.0003**	0.0013	−0.0007	0.0004	0.0041^*^
		**0.50**	0.0026	−0.0048^*^	0.0007	−0.0023	0.0014	**0.0006**

The table shows the bias (distance to the true cutpoint θ) for all simulation scenarios for method E1 (min AIC). *n*_*obs*_, the number of observations (for example, the number of patients); *pc*_*t*_, the total censoring in percent; *pc*_*f*_, the censoring before the end of the follow-up time in percent of total censoring *pc*_*t*_. The smallest bias per row is marked in bold, whereas the highest is marked with an asterisk (*).

### Empirical standard error (EmpSE) and mean squared error (MSE)

In an evaluation, in which only the true cutpoint *θ = *0.2 or *θ = *1.2 is distinguished, method E1 has the lowest EmpSE and MSE for both *θ*. The largest EmpSE for *θ = *0.2 is obtained using the E2 method. At a *θ = *1.2, methods D1 and D2 provide the largest EmpSE (Table 2). The largest MSE, regardless of the cutpoint, occurs using method E2. Sorting the methods used from lowest to largest EmpSE, for *θ = *0.2, the results are: E1, C2, B, F, C1, D2, D1, and E2. For *θ = *1.2, as for the bias, the order is also different for the EmpSE: E1, F, E2, B, C2, C1 and D1, D2. Therefore, the relative precision gain with method E1 as a reference shows, that method E2 for θ = 0.2, along with methods D1 and D2 for θ = 1.2, each have the greatest precision loss compared to the reference (E1). All methods indicate that the greater the deviation of the true cutpoint *θ* from the median, the larger the EmpSE becomes.

### Illustrations and applications with clinical examples of data

**Fig 6:**
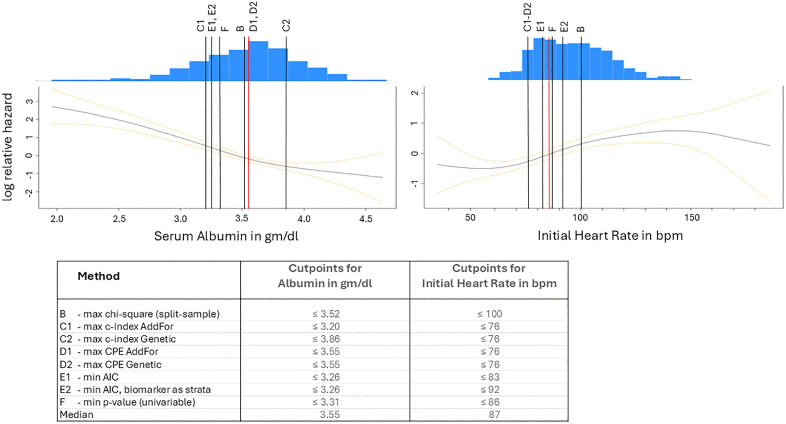
Combined log relative hazard and density plots with the estimated cutpoints as vertical lines. The vertical, numbered lines indicate the estimated cutpoints. Red lines represent the median. The lower part of the figure shows the cutpoints for albumin and initial heart rate for each method. bpm, beats per minute.

We also illustrate all methods on two freely available clinical data examples, which are available under a Creative Commons Attribution 4.0 International (CC BY 4.0) license. The first dataset consists of 312 patients with primary biliary cirrhosis (PBC) stages I to IV, all of whom took part in a study conducted at the Mayo Clinic [30,31]. The data was collected prospectively to evaluate the effectiveness of D-penicillamine in treating primary biliary cirrhosis. A liver biopsy was conducted initially to assess the histological stage. Out of the initial 312 patients, 125 died, resulting in a median trial duration of 39 months. Furthermore, 27 patients were either lost to follow-up or had undergone liver transplants. The remaining 160 patients were still alive and being monitored. According to Dickson and colleagues, we utilised relevant baseline variables for the prognosis of survival time [32]. The variables comprise: serum albumin (mg/dl), total serum bilirubin (mg/dl), age (years), and oedema. Prothrombin time was not included due to the significant variability inherent in the laboratory measurements for this parameter [33]. The cutpoints were determined for the biomarker serum albumin. Serum bilirubin, age, and oedema were used as covariates in the multivariable Cox regression model. The follow-up time was measured in months, and the status was indicated as deceased or alive. The distribution of serum albumin is illustrated in figure 6 (Fig 6). This figure further indicates that low albumin levels correlate with an increased risk in the studied population.

The second freely available dataset, from the R package smoothHR referred to as WHAS500, contains data from 500 patients who participated in the Worcester Heart Attack Study [34]. The Worcester Heart Attack Study aimed to identify factors related to trends of overall survival after hospital admission for acute myocardial infarction over time. From this dataset, we used the variables Initial Heart Rate (HR) in beats per minute (bpm), age at hospital admission in years (age), sex, body mass index (BMI) in kg/m^2^, follow-up time (time), and vital status at last follow-up (status). Cutpoints were estimated for the biomarker initial heart rate. The mean of HR is 87, and the median is 85. For distribution of HR see Fig 6. Covariates in the multivariable estimation model are age, gender, and BMI.

### Results of the cutpoint estimations

Cutpoints for serum albumin in the first dataset and for Initial Heart Rate in the WHAS500 dataset could be estimated with all multivariable methods (B, C1, C2, D1, D2, E1, E2) as well as with the univariable approach, method F (Fig 6).

The results for the CPE-based methods (D1 and D2) are identical (≤ 3.55 gm/dl for serum albumin and ≤ 76 bpm for HR). For initial heart rate, the cutpoints for methods C1, C2 and D1, D2 are equal. Methods E1 and E2 provide the same cutpoint for serum albumin, but different cutpoints for heart rate. Except for the cutpoint for albumin according to method C2, all cutpoints fall clearly within biomarker ranges where the relative hazard does not remain constant.

## Discussion

A review of the literature was conducted prior to our simulation study. Through this, we aimed to identify publications on methods for determining cutpoints within the multivariate Cox regression framework. Four studies were identified, covering a total of eight different methods. These methods were chosen, since they were the only ones to be found in the literature.

Under simulation, there were only 0.022 to 0.074 percent missing values for some methods, so we could compare all the results without any restrictions. We couldn’t determine, however, why a few scenarios were aborted of the R-package CatPredi. All procedures that have been reviewed are biased. In most simulated scenarios, all methods tend towards the median (Tables 2–4, Figs 4 and 5). In our simulation, it has been shown that methods A (max *χ*^*2*^) and E1 (min AIC), which lead to the same results, have both the lowest bias and the lowest EmpSE. This confirms the study by Mazumdar et al., who found that the univariable method F is inferior to method A [22]. Method B (max *χ*^*2*^ split sample) performs worse than the univariable method F, if the cutpoint *θ* is further away from the median. This is the case both in terms of the bias and the EmpSE. Method E2, in which the biomarker to be dichotomised is used as a strata variable in the Cox regression, performs well in this respect as the true cutpoint is close to the median. However, if the cutpoint is not the median, this method (E2) has the most considerable bias. For the E2 method, the BIAS in some scenarios is so large that the calculated cutpoint in our simulation deviates by more than one standard deviation from the theoretical cutpoint in some cases. If the calculated cutpoint is used to stratify patients or make treatment decisions, it could result in over 30% of patients being incorrectly classified or receiving the wrong treatment. The other multivariate methods exhibit a low BIAS on average (Fig 3). However, the BIAS of these methods becomes clinically relevant if the cutpoint is further from the mean, in cases where the sample size is smaller, and if the censoring rate is higher (Fig 4). Under these circumstances, the other methods also exhibit a BIAS that approaches or exceeds one standard deviation. This could lead to a relevant misclassification rate of patients in a clinical setting. As shown in the clinical examples, the cutpoints for both albumin and heart rate vary considerably depending on the chosen method. When calculated with method B, the cutpoint for heart rate is ≤ 100 bpm, which clearly differs from the cutpoint calculated with method E1 (HR ≤ 83 bpm) (Fig 6). This difference between methods B and E1 arises from the smaller sample size in the split-sample approach. This would mean that if patients were classified in a clinical study, according to method B, 125 patients would be placed in the above-cutpoint group, while 265 would be placed in that group using method E1. Therefore, using method B, which shows a higher BIAS in our simulation than method E1 (Fig 3), could lead to more than 100 of the 500 total patients being misclassified. By contrast, in both the albumin and heart rate examples, it makes little clinical difference whether the cutpoint is based on method D1 or D2, as the cutpoints are the same for both (HR ≤ 76 bpm and albumin ≤ 3.55 gm/dl).

When using method C2 (c-index Genetic), the bias and EmpSE are lower than with methods C1, D1, and D2. Barrio et al. pointed out, that the genetic algorithm is supposed to perform better than the AddFor algorithm when estimating two cutpoints of a biomarker [19]. Our simulation did not demonstrate that the Genetic algorithm generally outperforms the AddFor algorithm. Of these four methods, only if the true cutpoint is close to the median, method C2 is superior to the univariable approach F regarding bias and EmpSE. Nevertheless, the difference between these four methods (C1, C2, D1, and D2) is minimal. This can also be somewhat observed in the practical applications.

Table 2 shows that the MCSEs for the bias and EmpSE are so low, that this was no obstacle to meaningfully interpret our obtained values for the bias and EmpSE (Table 2). The EmpSE and MSE do not show the same patterns when compared to the performance measures used in each method. Morris et al. have pointed out that the MSE is more sensitive to *n*_*obs*_ than the EmpSE [23]. That is why we focused primarily on the Bias and EmpSE.

The initial selection of the regression coefficients (*β*_*X*_, *β*_*Z`*_) was based on simulation studies from Barrio et al. and Mazumdar et al., which used hazard ratios ranging from 0.5 to 4.0 [19,22]. Before running simulations, we performed pre-tests to ensure that the betas were large enough to generate significant omnibus tests of model coefficients in Cox regressions (p < 0.05), even at a sample size of n = 250. However, the specific beta values chosen may limit the generalisability of our findings, as clinical studies often report hazard ratios closer to 1 for certain biomarkers. With respect to all-cause mortality in cancer patients for example, Lena et al. reported hazard ratios of 0.91 for haemoglobin (per 1 g/dL) and 0.99 for the estimated glomerular filtration rate (GFR) (per 1 mL/min/1.73 m^2^) [35]. Since betas were held constant in our simulation, we cannot evaluate in what way the bias depends on the beta values.

We used only one cutpoint in our simulation. In contrast to Barrio et al., who investigated the methods C1, C2, D1, and D2 for the case of one, two, and three cutpoints per biomarker, we cannot make a statement for a scenario with several cutpoints [19]. Besides, the existence of one or even several cutpoints in real data sets may be unknown.

Utilising the R-package CatPredi, the computation of a cutpoint employing the C2 or D2 method requires approximately 44 or 87 seconds, if N = 500 and three covariates are included. In contrast to that, under identical conditions and hardware specifications (Windows 11, x64-based, CPU 2.40 GHz, 8 cores), the R-package cutpoint needs approximately 12 seconds for method E1. In particular, the methods C2 and D2 require longer computing times, as Barrio et al. have also pointed out [19]. This makes it difficult to carry out simulations with these methods, as they are associated with long computing times. However, the time component should hardly be relevant for determining individual cutpoints.

Our goal was not to determine the hazard ratios of the dichotomised biomarker *X* and associated p-values. Method B (max *χ*^*2*^ split-sample), used in the simulation study, offers the possibility to determine hazard ratios and the corresponding p-values on the other half of the data. However, method B has a substantially higher bias and negative precision gain in comparison with methods A and E1. Therefore, we recommend, as well as [22], the use of the cross-validation approach, as the entire data set is used to determine the cutpoint.

As the determination of cutpoints in the practical examples and the simulation study has demonstrated, the cutpoints can vary strongly depending on the method used. Therefore, examining spline plots and consulting with a medical specialist can be beneficial. Nevertheless, if the research areas have not been established for long, medical specialists or physicians may have limited knowledge to contribute to the decision in favour of a cutpoint. However, the dichotomisation of a metric variable should only be carried out if it cannot be avoided, as it is associated with a significant loss of information and power [15,16,22].

## Conclusions

Our simulation has shown that methods maximising the chi-square statistic or minimising the AIC, respectively, perform better than the univariable method using the minimum p-value approach and outperform methods based on the c-index or CPE. It remains unclear whether these two methods (A and E1) perform just as well when there are two or more cutpoints per biomarker. The method in which the dichotomised variable is used as a strata variable in the Cox regression model, is potentially associated with large bias.
